# FOXM1 coming of age: time for translation into clinical benefits?

**DOI:** 10.3389/fonc.2012.00146

**Published:** 2012-10-15

**Authors:** Muy-Teck Teh

**Affiliations:** Centre for Clinical and Diagnostic Oral Sciences, Barts and The London School of Medicine and Dentistry, Queen Mary University of LondonLondon, UK

**Keywords:** FOXM1, cancer biomarkers, epigenetic markers, cancer stem cells, personalised medicine

## Abstract

A decade since the first evidence implicating the cell cycle transcription factor Forkhead Box M1 (FOXM1) in human tumorigenesis, a slew of subsequent studies revealed an oncogenic role of FOXM1 in the majority of human cancers including oral, nasopharynx, oropharynx, esophagus, breast, ovary, prostate, lung, liver, pancreas, kidney, colon, brain, cervix, thyroid, bladder, uterus, testis, stomach, skin, and blood. Its aberrant upregulation in almost all different cancer types suggests a fundamental role for FOXM1 in tumorigenesis. Its dose-dependent expression pattern correlated well with tumor progression starting from cancer predisposition and initiation, early premalignancy and progression, to metastatic invasion. In addition, emerging studies have demonstrated a causal link between FOXM1 and chemotherapeutic drug resistance. Despite the well-established multifaceted roles for FOXM1 in all stages of oncogenesis, its translation into clinical benefit is yet to materialize. In this contribution, I reviewed and discussed how our current knowledge on the oncogenic mechanisms of FOXM1 could be exploited for clinical use as biomarker for risk prediction, early cancer screening, molecular diagnostics/prognostics, and/or companion diagnostics for personalized cancer therapy.

## INTRODUCTION

The human Forkhead Box M1 (FOXM1) protein, belongs to a winged-helix transcription factor family (Jackson et al., 2010), was first identified as a mitotic-phase phosphoprotein (MPP2) from a cervical cancer HeLa cell line (Westendorf et al., 1994) and its gene structure later mapped to chromosome 12p13.3 consisting of 10 exons, two of which are alternatively expressed thereby producing three alternatively spliced mRNA isoforms (Korver et al., 1997; Ye et al., 1997). Although all three protein isoforms of FOXM1 can bind to DNA, only FOXM1B and FOXM1C (isoform 2) were shown to be transcriptionally active (Korver et al., 1997; Ye et al., 1997). FOXM1A was found to be transcriptionally inactive due to the presence of an inhibitory exon (A2) in the C-terminal of its transactivation domain (Ye et al., 1997). Although FOXM1B was found to be the only isoform showing cell cycle-dependent mRNA expression in two different human cell lines (Gemenetzidis et al., 2010), it was not clear if this was due to splicing variations or other mechanisms, hence, further studies are required to clarify this issue. Most studies to date focused on FOXM1B and FOXM1C due to their transactivating roles in cell cycle which inadvertently led to the lack of studies on the inactive isoform FOXM1A whereby its role in cell cycle remains unknown. Given the presence of three FOXM1 isoforms in human, two in rat and one in mouse, the functional significance and interactions amongst the three human FOXM1 isoforms deserves further investigations.

## FOXM1 IN HUMAN CANCER

Early studies have demonstrated that FOXM1 was upregulated in a variety of human epithelial cancer cell lines (Ye et al., 1997) and that the high risk human papillomavirus (HPV) type 16 E7 oncoprotein interacted with Foxm1 to promote malignant transformation in cultured rat embryo fibroblasts (Luscher-Firzlaff et al., 1999). However, it was not clear whether FOXM1 had a causative role in human cancer *in vivo* until the first evidence demonstrated that FOXM1 was upregulated in basal cell carcinomas (Teh et al., 2002), one of the most common human skin cancers worldwide. FOXM1 was a downstream target of an oncogenic Sonic Hedgehog signaling pathway via a glioma family zinc finger transcription factor 1 (Gli1) in basal cell carcinomas (Teh et al., 2002). Subsequent studies revealed that FOXM1 was aberrantly upregulated in the majority of human cancers (Myatt and Lam, 2007; Wierstra and Alves, 2007) which include liver, breast, prostate, lung, brain, colon, pancreas, testis, bladder, kidney, ovary, uterus, cervix, oral (Gemenetzidis et al., 2009; Waseem et al., 2010), stomach (Li et al., 2009), blood (acute myeloid leukemia; Nakamura et al., 2010), cutaneous melanoma (Huynh et al., 2011), thyroid carcinoma (Ahmed et al., 2012), nasopharyngeal carcinoma (Chen et al., 2012), and esophageal cancer (Gemenetzidis et al., 2009; Hui et al., 2012).

Given a role in cell cycle, it is not surprising that FOXM1 plays a pivotal role in tumorigenesis. FOXM1 expression level has been shown in numerous types of human cancer to be dose-dependently correlated with tumor progression starting from cancer predisposition and initiation (Gemenetzidis et al., 2010; Jia et al., 2010; Teh et al., 2010), early premalignancy and progression (Gemenetzidis et al., 2009; Nakamura et al., 2010; Waseem et al., 2010; Huynh et al., 2011) to metastatic invasion (reviewed in Wierstra and Alves, 2007). Importantly, FOXM1 expression has been inversely correlated with poor prognosis in patients with oral squamous cell carcinoma (Chen et al., 2009), glioblastoma (Liu et al., 2006), breast cancer (Bektas et al., 2008; Martin et al., 2008), hepatocellular carcinoma (Sun et al., 2011; Xia et al., 2012), pulmonary squamous cell carcinoma (Yang et al., 2009), and colorectal cancer (Chu et al., 2012). Furthermore, emerging studies have shown that FOXM1 confers resistance to a wide variety of breast cancer chemotherapeutic drugs (reviewed in Wilson et al., 2011). Hence, it appears that FOXM1 is required and necessary in all stages of tumorigenesis and metastasis.

## FOXM1 IN STEM CELL FATE DETERMINATION AND CANCER INITIATION

Adult stem cells are responsible for tissue homeostasis and repair. However, due to their inherently high clonogenic potential and plasticity, stem cells are susceptible to oncogenic selection rendering these cells ideal targets for cancer initiation. In rare occasions, tumors may arise spontaneously and rapidly without sequential accumulation/selection of oncogenic mutations through a catastrophic genomic rearrangement event, namely chromothripsis (Liu et al., 2011; Stephens et al., 2011; Crasta et al., 2012). Nevertheless, it is generally accepted that the majority of malignancies are initiated by stem cells which accumulate and propagate oncogenic mutations through clonal evolutionary selection.

Emerging evidence have indicated that FOXM1 plays an important role in maintaining stem cell renewal through pluripotency genes Oct4, Nanog, and Sox2 in mouse (Xie et al., 2010; Tompkins et al., 2011; Wang et al., 2011). A recent mouse model study established a key role for FOXM1 in cell fate determination. This study showed that FOXM1 regulated mammary luminal cell fate by modulating the expression of GATA-3, a key regulator of breast luminal epithelial differentiation (Carr et al., 2012). Furthermore, FOXM1 has been shown to transactivate an epithelial stem cell marker keratin 15 (KRT15) gene in human keratinocytes (Bose et al., 2012).

**FIGURE 1:**
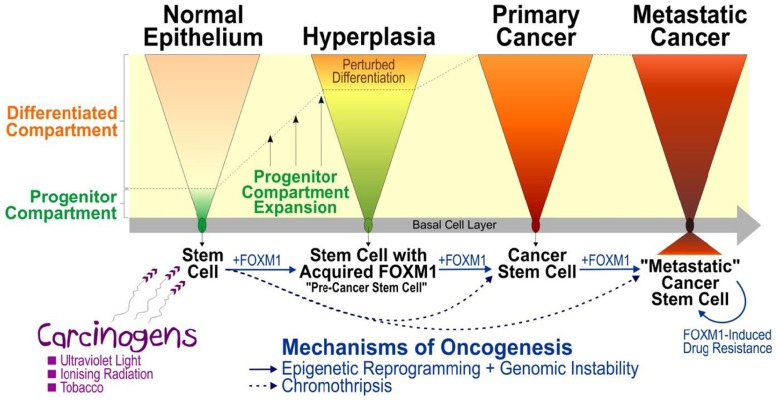
**A model mechanism illustrating the role of FOXM1 in human epithelial cancer initiation, progression, and metastasis**. Multiple lines of evidence have suggested that carcinogens (such as ultraviolet light, ionizing radiation, tobacco, etc) exposure causes activation of FOXM1 which triggers aberrant expansion of “pre-cancer stem cells” through perturbation of epithelial differentiation, producing a premalignant hyperplastic phenotype (Gemenetzidis et al., 2010). Activation of genomic instability (e.g., through activation of CEP55 leading to mitotic instability; Gemenetzidis et al., 2009) and epigenetic reprograming (e.g., through deregulation of HELLS causing chromatin remodeling and altered genomic methylation) triggered by aberrant expression of FOXM1 (Teh et al., 2012) may predispose cells to further mutations thereby driving oncogenic progression and subsequent metastatic invasion. Due to the complexity of genomic instability and epigenetic reprograming activated by FOXM1, this model may explain the heterogeneity found in tumor whereby the initial molecularly distinct “pre-cancer stem cells” undergo constant adaptive evolutionary changes to produce “cancer stem cells” and subsequently “metastatic cancer stem cells” during the course of cancer progression. An alternative mechanism involving catastrophic genomic rearrangement, chromothripsis, has been shown to produce tumor directly and rapidly from normal cells without the need for sequential accumulation of oncogenic mutations (Liu et al., 2011; Stephens et al., 2011; Crasta et al., 2012).

It has been demonstrated that environmental (e.g., sun exposure) and carcinogenic factors (e.g., tobacco use, etc.) can cause aberrant expression of FOXM1 leading to cellular proliferation and promote oncogenic genomic instability in human cells (**Figure 1**). It has been shown that ionizing radiation, etoposide, or ultraviolet light-induced DNA damage leads to Chk2-mediated FOXM1 phosphorylation and its stabilization (Tan et al., 2007). Furthermore, repeated ultraviolet B irradiation on human keratinocytes enhanced FOXM1-associated genomic instability in the form of loss of heterozygosity (LOH) and copy number aberrations (CNA) perturbing genomic loci containing large number of genes, for example, epidermal growth factor receptor (EGFR), insulin-like growth factor 2 receptor (IGF2R), and insulin-like growth factor binding protein 1/3 (IGFBP1/3), which have been previously linked to oncogenesis of human squamous cell carcinoma (Teh et al., 2010). Similarly, nicotine has been shown to promote malignant transformation by enhancing FOXM1-associated LOH and CNA, whereby malignant cells bearing amplified CNV loci (10q23) containing a centrosomal protein CEP55 responsible for cytokinesis and a chromatin-remodeling helicase/stem cell factor HELLS known to regulate epigenetic reprograming (Gemenetzidis et al., 2009; Teh et al., 2012). The complexity of genomic instability and epigenetic reprograming activated by FOXM1 may therefore generate a highly heterogeneous population of mutant cells ready to adopt subsequent oncogenic insults which may explain the heterogeneity exists in many cancers. Taken together, these studies support a driver role of FOXM1 in cancer predisposition and initiation through perturbation of the genomic and epigenomic landscapes (Gemenetzidis et al., 2009; Teh et al., 2010, 2012).

It was unclear how normal healthy cells retain abnormal expression of FOXM1 following exposure to carcinogens. Using a well-established three dimension (3D) human organotypical tissue culture model system enabled us to study epithelial differentiation and renewal mechanism with high degree of similarity to human tissue regeneration *in vivo* (Gemenetzidis et al., 2010) without provoking ethical issues associated with human or animal subjects. This human organotypical culture study has provided the first direct evidence that FOXM1 regulates human adult epithelial stem cell fate (Gemenetzidis et al., 2010). Overexpression of FOXM1 in human keratinocyte stem/progenitor cells, but not in differentiating cells, significantly expanded the proliferative progenitor compartment by perturbing epithelial differentiation producing a hyperproliferative phenotype reminiscent of that seen in hyperplasia – a condition that carries a risk of malignant transformation depending on subsequent oncogenic hits. This finding indicates that FOXM1 hijacks the self-renewal properties of stem cells to initiate a premalignant condition sustained by molecularly distinct “pre-cancer” stem cells. The acquisition of aberrant expression of FOXM1 by normal stem cells may represent a key driver step in a multistep oncogenic evolutionary pathway (**Figure 1**).

Ectopic FOXM1 has been found to induce stem/progenitor compartment expansion by shifting the balance toward stem cell renewal whilst perturbing differentiation (Gemenetzidis et al., 2010; Bao et al., 2011; Kalin et al., 2011; Wang et al., 2011; Bose et al., 2012) and cause genomic instability in human cells through deregulation of mitosis and/or cytokinesis (Laoukili et al., 2005; Gemenetzidis et al., 2009). Moreover, aberrant FOXM1 expression also induces epigenomic perturbations through activation of a chromatin-remodeling helicase/stem cell factor HELLS (Gemenetzidis et al., 2009; Teh et al., 2012), activated epithelial–mesenchymal transition (reviewed in Wierstra and Alves, 2007; Gemenetzidis et al., 2010; Kalin et al., 2011) and induces DNA-repair/drug resistance pathways (reviewed in Wilson et al., 2011). Collectively, these findings illustrate diverse molecular mechanisms of how aberrant expression of FOXM1 may play pivotal roles in all stages of tumorigenesis from initiation to metastatic invasion.

## CLINICAL TRANSLATION

**FIGURE 2:**
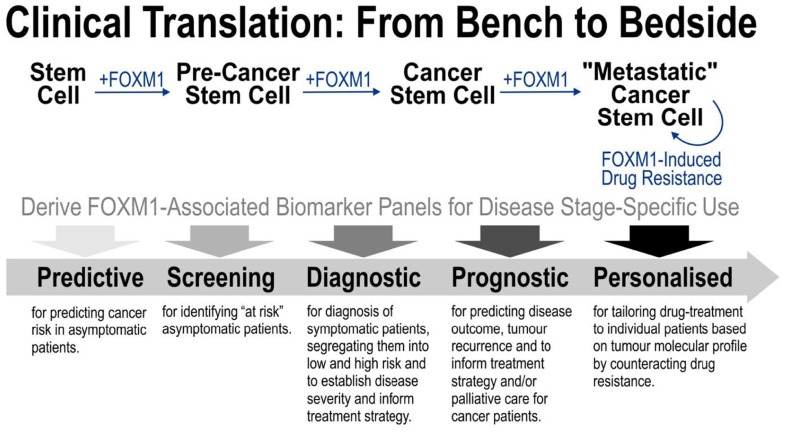
**Strategy for translating basic FOXM1 research into clinical benefits**. FOXM1 has been implicated in all stages from cancer initiation, progression, metastasis to drug resistance. Understanding the detail molecular mechanisms specific to each disease stages would be important to reveal stage-specific FOXM1-associated biomarker panels as illustrated in the diagram. Identification of these new stage-specific biomarkers paves way toward further research into developing more accurate cancer diagnosis/prognosis and better anti-tumor drugs.

Understanding the basic molecular mechanism of FOXM1-driven oncogenesis is prerequisite to exploitation for clinical benefits. Given that FOXM1 has been implicated in all stages from cancer initiation, progression, metastasis to drug resistance, FOXM1 is evidently a promising cancer biomarker. However, understanding the detail molecular mechanisms specific to each disease stages would be important to reveal stage-specific FOXM1-associated biomarker panels as illustrated in **Figure 2**. Furthermore, identification of such stage-specific biomarkers could in turn stimulate further research into finding new anti-tumor drugs with better specificity and efficacy.

## EPIGENETIC ALTERATIONS AS PREDICTIVE CANCER BIOMARKERS

Epigenetic programing plays a key role in cell fate diversification that involves mechanisms such as stem cell renewal, proliferation, differentiation, and aging (Baylin and Jones, 2011). DNA methylation is one of the fundamental epigenetic programing mechanisms whereby its heritable yet reversible methylome landscapes is able to produce diverse phenotypes from a single genome without altering its primary DNA sequence. Aberrant disturbance of the methylome landscape in normal cells is known to induce cancer formation (Tsai and Baylin, 2011). Given a presumed higher hierarchy of the epigenome over the transcriptome and proteome in terms of the Central Dogma of Molecular Biology, understanding aberrant epigenetic alteration involving DNA methylation is prerequisite to finding predictive, and/or early cancer biomarkers. Advances in detecting cell-free nucleic acids in cancer patients’ blood demand better nucleic acid-based biomarkers (Schwarzenbach et al., 2011a). The chemically distinctive and reversible properties of methylated DNA provide ample opportunities for clinical exploitation as nucleic acid-based biomarkers potentially detectable in non-invasive samples such as blood, buccal scrapes, or even saliva.

Given that FOXM1 was found to “brainwash” normal cells by reprograming the methylome and changing its landscape toward those found in cancer cells (Teh et al., 2012), the global epigenomic perturbations orchestrated by FOXM1 during pre-cancer initiation may therefore contain clinically exploitable predictive cancer biomarkers. In the attempt to identify pre-cancer or predictive cancer biomarkers accrued during aberrant cell proliferation induced by FOXM1, we have investigated this using genome-wide methylome arrays to identify FOXM1-orchestrated differentially methylated genes in primary normal human epithelial cells (Teh et al., 2012). A number of FOXM1-induced differentially methylated genes were identified, including SPCS1, FLNA, CHPF, GLT8D1, C6orf136, MGAT1, NDUFA10, and PAFAH1B3. These genes were also found to be differentially expressed in head and neck squamous carcinoma tumor samples compared to control normal tissues (Teh et al., 2012). However, in order to establish whether methylation profiles of these genes have any cancer predictive value, further longitudinal studies correlating pre-symptomatic patient data with subsequent disease outcome are required. Such predictive biomarkers would have tremendous clinical value for population screening to identify individuals with cancer predisposition or at risk of developing cancer. Increase public awareness, clinical surveillance and appropriate preventive interventions, such as behavioral or lifestyle changes, may significantly delay or even avert cancer initiation. In cases where cancer initiation could not be prevented, early detection of pre-cancerous lesions together with appropriate intervention can significantly improve patient’s quality of life, better treatment outcome and alleviate healthcare costs (Baron et al., 2010). For example, 5-year-survival rates of patients with head and neck cancer could be significantly improved from less than 20% to over 80% if patients were diagnosed and treated at early cancer stages (Haddad and Shin, 2008).

## FOXM1 TRANSCRIPTIONAL TARGETS AS DIAGNOSTIC AND PROGNOSTIC CANCER BIOMARKERS

Perturbed epigenome invariably leads to genomic instability (Baylin and Jones, 2011; Tsai and Baylin, 2011). Aberrant expression of FOXM1 has been shown to perturb both the human methylome (Teh et al., 2012) and induces genomic instability (Gemenetzidis et al., 2009; Teh et al., 2010). We have found that FOXM1-induced genomic instability leads to heritable genomic alterations which were potentially oncogenic (Gemenetzidis et al., 2009; Teh et al., 2010, 2012). As genomic instability precedes malignant conversion (Gemenetzidis et al., 2009; Teh et al., 2010; Baylin and Jones, 2011; Tsai and Baylin, 2011), these genomic alterations are thought to contain clinically relevant “cancer progression” biomarkers for diagnosis and prognosis. FOXM1-induced LOH, CNA, and/or resultant gene expression alterations may be exploited to determine disease aggressiveness or segregate between high and low risks patients. For example, LOH markers within cell-free DNA found in blood samples have been shown to be clinically valuable as diagnostic/prognostic markers in breast cancer patients (Schwarzenbach et al., 2011b). Extracellular RNA molecules released into the blood stream were surprisingly stable possibly protected by being packaged in exosomes. Emerging evidences have shown that the levels of specific cell-free mRNA in blood samples were exploitable for clinical use as prognostic biomarkers (Schwarzenbach et al., 2011a; Tzimagiorgis et al., 2011). Detection of target mRNA signatures in cell-free biofluids (such as blood, urine, saliva, etc.) may therefore represent a promising non-invasive method for cancer diagnosis and prognosis.

## FUTURE PERSPECTIVE

Pre-cancer initiation and multifaceted oncogenic roles of FOXM1 in myriad of human cancers (Myatt and Lam, 2007; Koo et al., 2012) render it a highly promising cancer biomarker for clinical exploitation. Recent advances have shown promising clinical use of multi-gene mRNA expression signature in tumor tissue samples for cancer risk stratification in patients with non-small cell lung cancer (Kratz et al., 2012), prostate (Cuzick et al., 2011), breast cancer (Kim and Paik, 2010), sarcomas, gastrointestinal stromal tumors, and lymphomas (Chibon et al., 2010). Hence, exploiting FOXM1 and its key oncogenic epigenetic and transcriptional targets as multi-gene panels would be superior over using a single biomarker for a complex disease such as cancer. Disease stage-specific cancer predictive, diagnostic, and prognostic biomarkers driven by FOXM1 require further discovery and validation studies on clinical specimens. Given the role of FOXM1 in mediating therapeutic drug resistance in cancer cells (Koo et al., 2012), FOXM1 pathway-based multi-biomarkers panel could be exploited for use as a personalized companion diagnostic tool to guide the best treatment strategy and improve drug treatment response. In summary, our scientific knowledge of FOXM1-driven oncogenesis presents multifaceted clinically exploitable opportunities ranging from cancer prevention, early diagnostics, and prognostics to personalized diagnostics and therapeutics.

## Conflict of Interest Statement

The author is listed as an inventor on a patent application at the World Intellectual Property Organisation filed by Queen Mary University of London pertaining to the use of a panel of biomarkers for cancer diagnosis.
